# The lived experience of renal cachexia: An interpretive phenomenological analysis

**DOI:** 10.1016/j.ijnsa.2024.100235

**Published:** 2024-08-22

**Authors:** Carolyn Blair, Joanne Shields, Robert Mullan, William Johnston, Andrew Davenport, Denis Fouque, Kamyar Kalantar-Zadeh, Peter Maxwell, Clare McKeaveney, Helen Noble, Sam Porter, David Seres, Adrian Slee, Ian Swaine, Miles Witham, Joanne Reid

**Affiliations:** a School of Nursing and Midwifery, Queen's University Belfast, MBC Building, 97 Lisburn Road, Belfast BT9 7BL, United Kingdom; b Regional Nephrology Unit, Belfast City Hospital, Belfast Health & Social Care Trust, Belfast, United Kingdom; c Renal Unit, Antrim Area Hospital, Northern Health & Social Care Trust, Antrim, United Kingdom; d Renal Arts Group Patient Ambassador, Queen's University Belfast, Belfast, United Kingdom; e UCL Department of Renal Medicine Royal Free Hospital University College London, London, United Kingdom; f Division of Nephrology, Dialysis and Nutrition, Hôpital Lyon Sud and University of Lyon, France; g Irvine Division of Nephrology, Hypertension and Kidney Transplantation, University of California, Irvine, CA, USA; h Centre for Public Health, Queen's University Belfast, Belfast, United Kingdom; i Department of Social Sciences and Social Work, Bournemouth University, Poole, United Kingdom; j Institute of Human Nutrition and Department of Medicine, Columbia University Irving Medical Center, New York, NY, USA; k Division of Medicine, Faculty of Medical Sciences, University College London, London, United Kingdom; l School of Human Sciences, University of Greenwich, Greenwich, United Kingdom; m AGE Research Group, NIHR Newcastle Biomedical Research Centre, Campus for Ageing and Vitality, Newcastle University, Newcastle Upon Tyne, United Kingdom

**Keywords:** Cachexia, Kidney disease, Health, End-stage kidney disease, Palliative care, Lived experience, Qualitative, Interpretative phenomenological analysis

## Abstract

**Background:**

Chronic kidney disease is common, affecting up to 13 % of the global population, and is predicted to become the fifth leading cause of 'life years lost' by 2040. Individuals with end-stage kidney disease commonly develop complications such as protein-energy wasting and cachexia which further worsens their prognosis. The syndrome of ‘renal cachexia’ is poorly understood, under-diagnosed and even if recognised has limited treatment options.

**Objective:**

To explore the lived experience of renal cachexia for individuals with end-stage kidney disease and the interrelated experiences of their carers.

**Design:**

This interpretive phenomenological study was designed to facilitate an in-depth exploration of how patients and carers experience of renal cachexia. To improve and document the quality, transparency, and consistency of patient and public involvement in this study the Guidance for Reporting Involvement of Patients and the Public-Short Format was followed.

**Setting:**

The study was conducted across two nephrology directorates, within two healthcare trusts in the United Kingdom.

**Participants:**

Seven participants who met the inclusion criteria were recruited for this study, four patients (three female, one male) and three carers (two male, one female).

**Methods:**

We employed a purposive sampling strategy. Data collection was conducted between July 2022 and December 2023. Interviews were semi-structured, audio-recorded, transcribed verbatim and analysed in six steps by two researchers using interpretive phenomenological analysis. Ethical approval was approved by the Office for Research Ethics Committees Northern Ireland (Reference: 22/NI/0107).

**Results:**

Analysis generated six group experiential themes: the lived experience of appetite loss, functional decline and temporal coping, weight loss a visual metaphor of concern, social withdrawal and vulnerability, the emotional toll of eating challenges and psychological strain amidst a lack of information about cachexia.

**Conclusion:**

This is the first qualitative study exploring the lived experience of renal cachexia for patients and carers. Our study highlights that psycho-social and educational support is urgently needed. Additionally, healthcare professionals need better information provision to help them to recognise and respond to the needs of this population. Further research is required to develop models of holistic support which could help patients and carers cope with the impact of renal cachexia and optimally manage this syndrome within the family unit.

**Registration:**

N/A.

**Key learning points**

**What is already known**

• Globally, cachexia is a severe but frequently under-recognised problem.
• Cachexia is associated with increased morbidity and mortality, contributing to lower quality of life, elevated levels of depression and higher rates of hospitalisation.
• There is limited evidence about the presence and impact of cachexia in end-stage renal disease, highlighting the need for further research in this area.

**What this paper adds**

• This is the first study to explore the impact of renal cachexia through an in-depth interpretative phenomenological analysis of the lived experience of patients and carers.
• This study highlights that psycho-social and educational support that recognises and responds to the needs of this population is urgently needed.
• Further research is required to develop models of holistic support which could help patients and carers cope with the impact of renal cachexia and optimally manage this syndrome within the family unit.

Alt-text: Unlabelled box

## 1 Background

Chronic kidney disease has emerged as a leading cause of worldwide mortality affecting 13 % of the global population, and is predicted to become the fifth leading cause of 'life years lost' by 2040 (KRUK 2023, Jager et al., 2019, Abdalla et al., 2015). Patients with chronic (and in particular those with end-stage) kidney disease are at increased risk of cachexia (wasting) which is considered the most severe stage of protein-energy wasting (Koppe et al., 2019), with mortality rates reported ranging from 20–40 % per year (von Haehling et al., 2016). In individuals with chronic kidney disease, this syndrome is known as ‘renal cachexia’ (Koppe et al., 2019, Reid et al., 2018); it is under-diagnosed, under-treated and under-researched (Reid et al., 2018, Reid et al., 2013, Reid et al., 2015, McKeaveney et al., 2021, McKeaveney et al., 2021, McKeaveney et al., 2020, Reid, 2014).

Alongside protein energy wasting, individuals with renal cachexia typically present with anorexia and muscular weakness (Reid et al., 2018, Gandolfini et al., 2019), with the highest prevalence of wasting among those receiving haemodialysis treatments (up to 75 %) (Reid et al., 2018). Renal cachexia is associated with adverse clinical outcomes including reduced quality of life, lower functionality and increased mortality (Mak et al., 2011, Slee et al., 2020). Cachexia is characterised by an ‘objective’ aetiology encompassing inadequate food intake, weight loss, inactivity, muscle wasting and metabolic derangements, inducing catabolism (Fearon et al., 2013, Schcolnik-Cabrera et al., 2017, Arends et al., 2021) and a ‘subjective’ aetiology including anorexia, early satiety, taste alterations, chronic nausea, distress, fatigue and loss of concentration (Arends et al., 2021). Intensive dietary support and dialysis can sometimes reverse deterioration in nutritional status for individuals with chronic kidney disease who have anorexia-induced insufficient energy intake (Koppe et al., 2019, Mak et al., 2011, Cano et al., 2007, Cheung et al., 2010). However, it is much more difficult to improve the nutritional status and body composition in individuals who have renal cachexia because of additional, profound metabolic alterations (Koppe et al., 2019, Mak et al., 2011, Cano et al., 2007, Cheung et al., 2010).

Patients with end-stage kidney disease in receipt of haemodialysis and their carers are widely known to experience psychological difficulties and social isolation (Hejazi et al., 2021, Shahgholian and Yousefi, 2018, Chiaranai, 2016, Hoang et al., 2018). Research indicates that addressing the issues related to quality of life can positively affect patient and caregiver outcomes (Hejazi et al., 2021, Shahgholian and Yousefi, 2018, Chiaranai, 2016, Hoang et al., 2018). However, little is known about the experience of patients with end-stage kidney disease in receipt of haemodialysis with cachexia and their carers (McKeaveney et al., 2020). Research on cachexia in other conditions is more advanced than that of renal cachexia offering valuable psychosocial insights and interventions that have significantly improved patient care and outcomes (Oberholzer et al., 2013, Amano et al., 2022, Wheelwright et al., 2016). Most prominently, evidence outlines that patients with cachexia and their carers can suffer from inter-related eating-related distress which causes profound disturbances in family dynamics (Oberholzer et al., 2013, Amano et al., 2022, Wheelwright et al., 2016). The intensity of burden from cachexia when patients are in receipt of haemodialysis treatment is, to date, unknown from the perspective of patients and carers highlighting a pressing need for exploration.

There is a dearth of knowledge regarding the lived experience of renal cachexia (McKeaveney et al., 2021, McKeaveney et al., 2020). Studies focused on the lived experience of cancer cachexia (Reid et al., 2009, Reid et al., 2010) and cardiac cachexia (Carson et al., 2022) observed that carers of these patients face multifaceted challenges predominantly because of reduced appetite and the central role food and eating in everyday life. In these studies, this dual perspective enriched these data and provided a holistic view of the lived experience of cachexia (Reid et al., 2009, Reid et al., 2010, Carson et al., 2022). This evidence suggests that the close and regular contact which informal carers have with patients experiencing cachexia places them in a unique position to observe and understand the day-to-day experiences which the patient themselves might not recognise or may underreport (Reid et al., 2009, Reid et al., 2010, Carson et al., 2022). Through sharing observations for example, about the patient's mood and coping mechanisms and reporting on their efforts to care this helps to develop a complete picture of the caregiving dyad (Reid et al., 2009, Reid et al., 2010, Carson et al., 2022). When triangulated with the patients’ experience, carers enhance insight into the emotional and psychological toll of the disease which is essential in an under-recognised syndrome such as renal cachexia.

Despite the critical importance of addressing renal cachexia, no studies have specifically investigated the lived experience of these patients and their carers. Our study is the first to explore the impact of renal cachexia through an in-depth interpretative phenomenological analysis of the lived experience of patients and carers, through which we aimed to inform practice and targeted interventions to meet service user needs.

## 2 Methods

### 2.1 Research aim

The aim of this study was to explore the lived experience of renal cachexia for individuals with end-stage kidney disease and the interrelated experiences of their carers.

### 2.2 Design

Interpretive phenomenological analysis was used to facilitate an in-depth exploration of how patients with renal cachexia and their carers make sense of their existence within the context of their world (Smith, 2009). Heidegger's approach to phenomenology was used due to its emphasis on interpretation, contextuality, and existential concerns (Horrigan-Kelly et al., 2016, Heidegger, 1962). From a Heideggerian phenomenological approach, to interpret meaning the researcher must delve beyond surface-level descriptions of experiences and explore deeper existential dimensions that inform individuals' perceptions, actions, and interactions (Heidegger, 1962). To do this, a ‘double hermeneutic’ dialogue between the researcher and the participant with self‐awareness and reflexivity was required (Shaw, 2010). Smith and Osborn (Smith and Osborn, 2015) explained this process as one in which “the researcher is trying to make sense of the participant trying to make sense of what is happening to them” (p. 10). In phenomenological inquiry the experience must be recalled in such a way that the essential aspects of the experience are retrieved or brought back through the description of those who have experienced it (Van Manen, 2023). Therefore, the world of the lived experience, should be both the source and the object of the phenomenological research (Van Manen, 2023). Thus, involving carers in phenomenological research is recognised as a valid source of data and in this case vital to obtain a complete understanding of this phenomenon. Interpretive phenomenological analysis has been evidenced to effectively uncover how cachexia affects patients' and carers’ identities, their sense of self, and their relationships with others (Reid et al., 2009, Reid et al., 2010) and was the most suitable method to permit interpretation of the complex interactions in the experience of renal cachexia.

### 2.3 Setting

The study was conducted across two nephrology directorates, within two healthcare trusts in the United Kingdom.

### 2.4 Inclusion and exclusion criteria

The inclusion and exclusion criteria for participants are defined in Table 1. The operational definition of ‘renal cachexia’ in this study is based on Evans et al. (2008) generic definition of cachexia in chronic illness. Any individual who displayed secondary causes of cachexia, where their weight loss resulted from a clinically explainable reduced oral intake rather than the metabolic processes associated with cachexia, were excluded. Excluding individuals on these grounds reflects current research practice, which acknowledges that weight loss in cachexia is caused by more than just a simple reduction in food intake (Arends et al., 2021). The operational definition of ‘carer’ in this study is reflected in the inclusion criteria as a lay individual (i.e., non-healthcare professional who is seen as integral to the supportive journey for the individual living with renal cachexia, has regular (>5 times per week) face-to-face contact with them and is identified by the individual as their carer (Table 2, Table 3).

**Table 1:** Inclusion and exclusion criteria.

**Renal Population**	
**Inclusion Criteria**	**Exclusion Criteria**
18 years of age and over	Under 18 years of age
Are living/nursed at home	Are living/nursed as an inpatient in hospital or a resident in a care home.
Have a confirmed diagnosis of end-stage kidney disease and in receipt of haemodialysis	Are not receiving dialysis.
Haemoglobin (<12 g/dl)	Haemoglobin (>12 g/dl)
To be included in the study, participants will have:oedema-free weight loss of at least 5 % in 12 months or less. In cases where weight loss is not documented a BMI <20.0 kg/m^2^ is sufficient; plus 3 of the following • fatigue - defined as physical and/or mental weariness resulting from exertion; an inability to continue exercise at the same intensity with a resultant deterioration in performance; • anorexia - limited food intake (i.e. total caloric intake less than 20 kcal/kg body weight/d, <70 % of usual food intake) or poor appetite; • abnormal biochemistry: – increased inflammatory markers (CRP>5.0 mg/l), IL-6 >4.0 pg/ml) – low serum albumin (<3.2 g/dl) (Evans et al., 2008). Criteria will be confirmed through the clinical gatekeeper.	Experiencing weight loss due to other physical causes. Any participant who displays secondary causes of renal cachexia will be excluded for example: malabsorption (i.e. prolonged nausea or vomiting; oesophageal blockage; bowel obstruction; persistent diarrhoea), starvation, primary depression, hyperthyroidism. Criteria will be confirmed through the clinical gatekeeper.
Be alert and mentally competent (as assessed by a member of the multi-disciplinary team)	Cognitive impairment (as assessed by their consultant or health care team)
Have the ability to provide informed consent, read and write English	Non-English speaking
Able/willing to be involved.	Are not able/willing to be involved.
**Significant Other**	
**Inclusion Criteria**	**Exclusion Criteria**
18 years of age and over	Under 18 years of age
Have face-to-face contact with the individual in the renal population more than 5 times per week	Does not have face-to-face contact with their significant other more than 5 times per week
Identified by the individual in the renal population as their significant other	
Alert and mentally competent (self-assessment)	Cognitive impairment (as assessed by their consultant or health care team)
Have the ability to provide informed consent, read and write English	Non-English speaking
Able/willing to be involved.	Are not able/willing to be involved

**Table 2:** Participant characteristics.

**Patients**				
**Patient participant ID**	**P1**	**P2**	**P3**	**P4**
1. Age:	84 years	67 years	50 years	60 years
2. Gender (identifies as):	Male	Female	Female	Female
3. Dialysis vintage (years):	Five years	Two years	Three years	Two years
**Carers**				
**Carer participant ID**	**C1**	**No consenting carer**	**C2**	**C3**
1. Gender (identifies as):	Female	N/A	Male	Male
2. Relationship to patient	Wife	N/A	Husband	Husband

**Table 3:** Interview schedule.

**Opening Questions**	
I am interested to hear about the weight loss you have / your loved one has had; can you tell me about it?	
How would you describe your / your loved one's experience of weight loss?	
**Area of Interest**	**Follow-up Questions**
Thoughts	What are your thoughts about the weight loss you / your loved one have/has had?
Emotions	How does your / your loved one's weight loss make you feel?
Perception of self	How does your / your loved one's weight loss affect how you/they see yourself/themselves?
Social perception	How does your / your loved one's weight loss affect your social relationships with your family and friends?
Everyday impact	How does your / your loved one's weight loss affect your everyday life?
Long-term impact	What does this weight loss mean for you now?
Lived expertise	Given your experience, what advice would you give to family, friends and staff involved in caring for those with the same condition in the future?
**Additional Prompts**	
Could you tell me about…, How do you feel about…, What does it mean…, What do you think…, Can you tell me a little more about…, How do you see…	
**Debriefing Question**	
Is there anything else you would like to say or talk about that I haven't asked you today?	

### 2.5 Sampling

Interpretive phenomenological analysis is an idiographic approach which involves a detailed analysis of each case to produce ‘thick’ interpretative accounts of a small number of participants as opposed to a ‘thinner’ report of a larger sample (Smith, 2009). Smith (2009) stated that between three and 10 participants can represent an adequate sample, depending upon the depth of these data. The focus of our study was centred on the micro-analysis of individual experiences and the close analytic reading of seven participant's words (Pietkiewicz and Smith, 2014, Sim et al., 2018, Smith and Nizza, 2022, Nizza et al., 2021). Purposive and intensity sampling was used to select four willing individuals with renal cachexia and three of their carers (Smith, 2009, Quinn Patton, 2002) (Table 2). The age ranges of the patients (three female and one male) ranged from 50 - 84 years of age, all had been in receipt of haemodialysis for between two and five years. Two of the carers were the husbands of the patients and one was a wife. One carer did not contact us regarding willingness to find out more about the study therefore did not participate in an interview.

### 2.6 Recruitment procedure

The recruitment procedure was promoted through consultant nephrologists across two nephrology directorates, in the two recruiting healthcare trusts within the United Kingdom. Individuals with renal cachexia were identified and invited by a member of the clinical care team to participate when attending for their routine haemodialysis treatment. Individuals with renal cachexia were provided with an information pack, including an invitation letter, participant information sheet, and contact form which included a request for contact details. In receipt of the contact form an experienced female qualitative post-doctoral researcher (CB) had a conversation with prospective participants to explain the study, build rapport and provide assurance of having over 10 years’ experience of conducting qualitative research. Only participants who provided informed consent, met the stipulated inclusion criteria were approached to take part in this study and only carers of those individuals who took part in the research study were approached (Poole and Froggatt, 2002). Therefore, it was possible for a patient to be included in the study without a carer, however the reverse was not the case.

### 2.7 Data collection

**Fig. 1:**
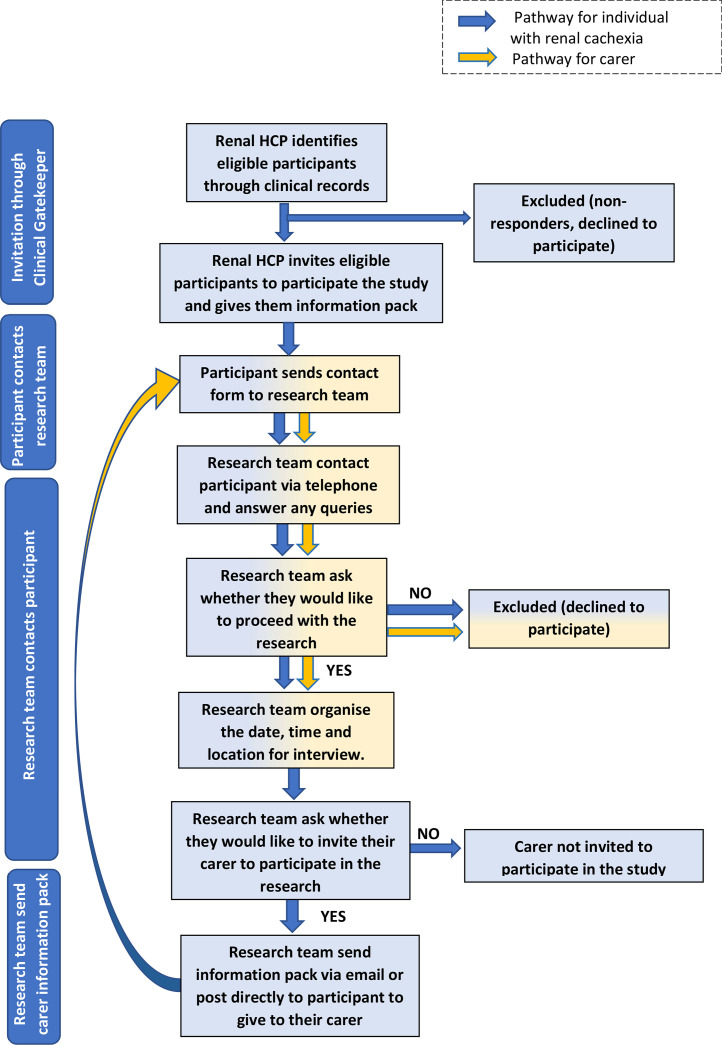
Recruitment flow chart.

One semi-structured interview was conducted per participant, the suitability of this data collection technique in phenomenological research is delineated in the literature (Smith, 2009). Prior to the commencement of interviews, signed informed consent forms were received via email or post. Individuals with cachexia and their carers participated in face-to-face interviews (n=2), telephone interviews (n=4) and online interview (n=1) alone via Microsoft Teams Version 1.7 at their homes. A lone working policy was implemented for domiciliary interviews. Audio was recorded via a Dictaphone and voice files deleted after transcription. The mean time for each interview was 45 minutes. Given the frailty of this patient population, a longer interview period would not have been possible Fig. 1.

### 2.8 Interview guide

An interview guide based on interpretive phenomenological analysis (Smith, 2009) was developed for the semi-structured interviews (Table 3). In keeping with Heidegger's approach, the questions were designed with contextualisation as a central focus (Horrigan-Kelly et al., 2016). The interview guides used for both individuals with renal cachexia and their carers contained a flexible format and were based on our literature review. Each participant was asked similar opening question based on the lived experience of weight loss, the term weight loss was used instead of cachexia as research suggested that patients would not be familiar with this terminology (Reid et al., 2009, Reid et al., 2010, Carson et al., 2022). The subsequent interview questions were in seven areas of interest on topics based on thoughts, emotions, perception of self, social perception, everyday impact, long-term impact and lived expertise. These questions were largely participant-driven and open ended as each participant responded to the questions according to their experiences in an unstructured manner (Smith, 2009). This allowed room to phrase questions spontaneously, to probe, clarify and reflect. To conclude each interview, an open question was asked to gain crucial data that would have otherwise been lost and gave an opportunity for interviewees to share anything that they deemed to be important (Morse and Field, 1996).

### 2.9 Analysis

**Fig. 2:**
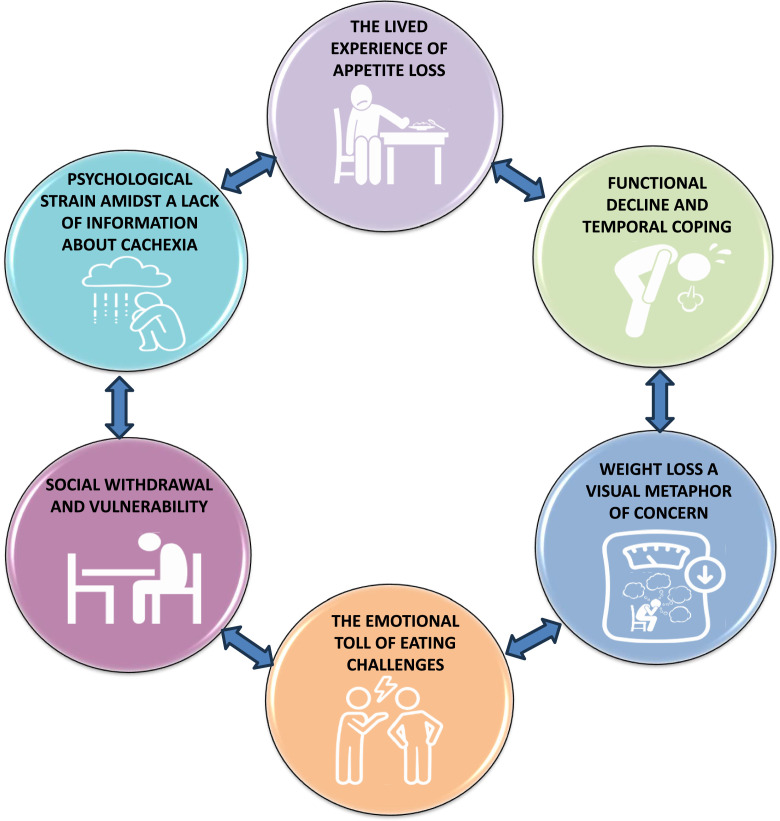
Six group experiential themes illustrating the lived experience of renal cachexia.

Aligned with the ideographic nature of interpretive phenomenological analysis (Smith, 2003) semi-structured interviews were used to obtain data which reflected the texture and nuances arising from the detailed exploration of each individual participant's subjective lived experience. To enhance reflexivity, throughout the study, from conception to dissemination, the researcher (CB) wrote reflective diary notes immediately after the interview to record observations and reflections, for example, non-verbal communication (Burnard, 1994) and other thoughts and comments of potential significance (Shaw, 2010, Vicary et al., 2017). This data was added to verbatim transcripts at the initial stage of analysis. We followed the six steps, as outlined by Smith et al. to identify group experiential themes (Smith and Nizza, 2022, Smith, 2003). Two members of the research team (CB and JR) independently worked through steps one to five, compared personal experiential themes and then worked collectively in step six to define the group experiential themes. Firstly, the initial interview was listened to multiple times, transcribed, and read multiple times. Secondly, areas of potential significance were highlighted on the transcript. Thirdly, statements which uncovered the lived experience of living with renal cachexia were clustered and subsequently developed into personal experiential themes. Fourthly, personal experiential themes were recorded and any correlations between them was noted. Fifthly, analysis continued with the remaining cases and all transcripts imported into NVivo 14 for data management (Edhlund and McDougall, 2019). Steps one to five were conducted with each interview transcript, before the cross-case analysis was conducted on personal experiential themes to identify group experiential themes (Smith and Nizza, 2022). As evolving clustering of group experiential themes developed, the initial transcript was re-checked to ensure any connections were unambiguous noting convergences and divergences to ensure the themes were representative (Smith and Nizza, 2022). Finally, in step six, when the analysis was complete a master list of group experiential themes was generated which was agreed by the two researchers (CB and JR) as a clear and accurate representation of the personal experiential themes across all cases. After, a visual representation of group experiential themes was created (see Fig. 2: Six group experiential themes illustrating the lived experience of renal cachexia).

### 2.10 Rigour

The data collection and subsequent analysis was stringently monitored through a transparent and well-documented methodological process to ensure rigour within the study. This exploratory qualitative study was designed using the consolidated criteria for reporting qualitative research (Tong et al., 2007) ( see supplementary file 1). Triangulating patient-reported experiences with carer observations enhanced the reliability and validity of the study findings, ensuring a more accurate representation of the lived experience of renal cachexia. We also used a process of confirming meaning by integrating reflective questioning within the interviews and using handwritten field notes of each interview which provided the necessary context for analysis (Given, 2008). To ensure that the analysis engaged with the complexities in the data set, two researchers (CB and JR) independently analysed data through five steps developing personal experiential themes, convergences and divergences were noted across studies and finally in the last step defined group experiential themes (Nizza et al., 2021, Smith, 2003). A patient and public involvement contributor (co-author) was a formal part of the research team from the beginning of the study and helped to guide the team in all patient-facing materials including interview questions. To improve and document the quality, transparency, and consistency of this involvement, we used the Guidance for Reporting Involvement of Patients and the Public-Short Format (Staniszewska et al., 2017) (see supplementary file 2). Dependability was fostered at all stages of this study, through triangulation, meaning confirmation, independent analysis and formal integration of a patient and public involvement contributor enabling future researchers to follow the same procedures (Lieblich et al., 1998).

### 2.11 Ethical considerations

Ethical approval for this study was granted by the Office for Research Ethics Committees Northern Ireland (Reference: 22/NI/0107) and data collected between July 2022 and Dec 2023. Essential aspects of good practice, including clear and sensitively written participant facing materials, voluntary participation, informed consent, confidentiality, and data protection procedures were employed as a minimum standard. Furthermore, the research team were experienced in qualitative research and designed the study to permit flexibility in questioning during interviews which allowed the emotions of participants to be sensed and questioning to be adapted appropriately. A distress protocol (Draucker et al., 2009) was used to ensure best practice as we were aware that participants may have experienced a high level of stress or emotional distress due to the sensitivity of the topic. Overall, the recruitment and data collection process were designed to reduce the burden on participants as much as possible.

## 3 Results

Analysis generated six group experiential themes for patients and carers: lived experience of appetite loss; functional decline and temporal coping; weight loss a visual metaphor of concern; social withdrawal, and vulnerability; the emotional toll of eating challenges; and psychological strain amidst lack of information in relation to cachexia. As illustrated in Fig. 2, these group experiential themes are bi-directional illustrating the complexity related to the lived experience of renal cachexia for patients and carers alike. In the narrative which follows, patients are identified by ‘P’ and carers by ‘C’ after their quotations.

### 3.1 The lived experience of appetite loss

Both carers and patients highlighted the significant changes in eating patterns and preferences including reduced appetite, a preference for “small meals” (P2) described as “a child's portion" (P1) and opting for starters or soup instead of full meals. Patients described the limitations imposed by early satiety, including a disconnection from embodied experiences such as the loss of a pleasurable sensory attitude toward food. The patients’ frustration regarding this aspect reflected a deep existential angst about their inability to engage fully with the world around them as they tried to make sense of the complex implications of the syndrome.

"Last time I was out I had a roast beef and I'd one bite and I couldn't eat any more …I couldn't face the idea of looking at food and knowing that I'd be wasting it. So, there's no point in getting me anything cause I'm not eating it." P2

Patients nostalgically reflected on memories of times when eating was different, when food brought pleasure and sustenance. These memories, tinged with sadness for patients, served as a contrast to their current experiences, which highlighted the profound changes brought about by renal cachexia and the challenges they faced in navigating early satiety.

“…away years ago, I used to love steaks, not a lot now, I eat half of it now and that's me full up” P1

Carers described an uncomfortable change to their everyday routine, planning for meals was described as previously an enjoyable activity which fostered bonding and connection; planning for meals was now a desperate search for anything their loved one might find appealing to eat. The everyday rituals and pleasures associated with eating no longer existed and a different more tumultuous dynamic was present. Given that the act of 'assisting to eat' served as a tangible expression of love for carers’ when their loved ones stopped eating foods they once cherished, carers were evidently distressed, frustrated and hope was diminished.

"...all of a sudden he stopped eating the things he always loved. I don't know if there's much more I can do with that, but I try.” C1

Despite the turmoil, carers empathetically expressed an awareness of the fear experienced by their loved ones when confronted with a large meal. They deemed a significant part of their caregiving role was to adapt and do everything possible to make eating possible and pleasurable for them.

"Even for her appetite, just wee tiny bits, eating often. Wee snacks instead of sitting down to a big meal. It seems to put people off if they have a big meal sitting in front of them." C2

Carers explained how their loved one's decline in appetite and loss of enjoyment in food had a major impact on their identity as a carer and necessitated a frantic change in how they expressed their care. Their desire to mitigate the effects of the syndrome fuelled a fixation on finding acceptable foods and doing everything possible to make eating pleasurable for their loved ones.

“Because you are trying to find something to cook for her that she will try to eat. And you are up and down trying to find different things. It's hard. It's hard to do sometimes.” C2

There was evidence of the emotional and physical toll of caregiving in the context of renal cachexia. Carers persistent efforts to meet the patients' needs despite the challenges they faced were acknowledged as difficult and complex, further emphasising their feelings of exhaustion and distress.

### 3.2 Functional decline and temporal coping

The experience of functional decline in the context of renal cachexia can be understood as a fundamental shift in the way patients related to their world and engaged with their everyday existence. The existential dependency caused by functional decline underscored the complex interplay between autonomy and dependency within the patients’ lived experience. Patients expressed feelings of vulnerability, helplessness, and loss of control over their own lives directly related to functional decline.

“I don't go out or anything. My daughter comes in the car and would take me up to shops like, but on my own, no I don't go out on my own anymore." P2

As patients grappled with the diminished capacities of their bodies, this disrupted their previously taken-for-granted modes of being and acting, as they came to terms with the limitations imposed by their illness.

"I don't have any energy at all, have no energy at all anymore.” P2

This disruption not only affected their ability to engage in activities they once enjoyed but also challenged their sense of self and identity. Patients struggled to reconcile their current state with their past experiences, leading to feelings of frustration, loss, and uncertainty.

"I feel like I can't join in the way other people would… I think I have a low grade of depression all the time, because these are the things I've got to think about. So, I don't seem terribly optimistic at times." P4

Similarly, carers had to adapt to a new mode of being in the world, characterised by the constant negotiation of uncertainty and unpredictability due to witnessing their loved one's functional decline. Carers were forced to confront the impermanence and fragility of life in the face of renal cachexia and found agency in focusing on the immediacy of the present moment.

"We just take one week at a time and see what happens. We don't plan too far ahead and stuff like that" C2

The forfeit of long-term planning in favour of adaptability and responsiveness to the ever-changing demands of caregiving for their loved one experiencing renal cachexia had significant consequences on their quality of life. The impact of functional decline on carers' leisure time and self-care revealed existential dimensions inherent in the carer-patient relationship. Carers’ sense of self and well-being were intricately linked to the needs and experiences of their loved one. As carers witnessed the progressive functional decline of their loved one due to renal cachexia, their leisure activities and opportunities for self-care were profoundly affected. Activities that were once shared and enjoyed together became inaccessible or had limited tenability due to functional decline. This disruption to their shared routines and leisure pursuits underscored the challenges faced by carers as they navigated the changing dynamics of their relationship.

Well, he would have went out and walked, we would have walked a bit which he can't do any more” C1

The carers’ sense of self was deeply intertwined with their role as a carer, and they often found themselves unable or unwilling to develop new forms of leisure activity and self-care in service of their caregiving responsibilities. This tension between caregiving and self-care highlighted the complex interplay between fear of upsetting their loved one and personal fulfilment within the carer's lived experience. As carers strived to meet the needs of their loved ones, they experienced feelings of guilt, frustration, and exhaustion as their own needs were, at times, suppressed in efforts to avoid further suffering for their loved one.

### 3.3 Weight loss a visual metaphor of concern

The visible manifestation of weight loss symbolised deeper worries for carers about disease progression, treatment efficacy, and overall health in caring for their loved one experiencing renal cachexia. Weight loss served as a tangible representation of bodily decline, increased fragility, and a visual reminder of impending mortality. In watching their loved one's decline, carers expressed worry, which was tainted with feelings of guilt as they questioned whether they could do anything more to alleviate the suffering of their loved ones.

“It's just hard to watch sometimes. She's losing so much weight, and you are just worried about her health and what she's going through and stuff” C2

Carers expressed fear at the thought of losing their loved one which illustrated the strong emotional bond and interconnectedness between patients and carers. It was evident that weight loss in the context of illness evoked anxiety and was a painful daily visual reminder for carers. Carers grappled with anxiety driven by the uncertainty of what lies beyond as their identity was primarily shaped by their caregiving role for their loved one. Additionally, this fuelled the anxious drive to make their loved one eat in efforts to prevent the progression of illness.

“You know it scares me, cause I don't know what I do without him… I can't just let him fall apart in front of me” C1

The evolving perceptions and concerns of both patients and carers reflected a shift in their interpretation of weight loss and an increasing awareness of bodily decline and mortality. Initially, patients and carers did not perceive weight loss as a significant issue, but over time, as they witnessed the decreasing weight and the erosion of physical vitality, it became a cause for concern.

“Well at first, I didn't really think it was anything to worry about. But it has been now. I just seemed to be getting less and less. There's nothing of me.” P3

The concern was embedded in the continual weight loss despite efforts to maintain weight, which resulted in a recognition of their lack of control over the trajectory of illness. This lack of control was subsequently channelled into vehement efforts to control the practical aspects of buying food, cooking and coaxing their loved ones to eat.

### 3.4 The emotional toll of eating challenges

Carers express a willingness to accommodate the patient's preferences by preparing and consuming foods that the patient likes, even if it's not the carer's favoured meals. By sacrificing their own desires to accommodate the patient's preferences, carers engage in a process of identity formation and meaning-making that is deeply intertwined with their role as carers. The tension over eating reflected a struggle between the desire to maintain and increase their loved ones’ weight and an understanding of the need to preserve their loved ones’ autonomy and preferences.

“I go for what he likes, sometimes you know he'll have a fry at night, and you know I'll have one too, even though I don't really like it.” C1

Carers demonstrated an awareness of the need for their loved ones to eat and would go to every length to coax them to eat. Carers’ expression of difficulty in finding food options for their loved one twinned with patients' interpretation of their carer trying to force them to eat was a manifestation of tension in this relational dynamic.

“But it is hard sometimes trying to find something for her to eat, that she will try to eat.” C2

“So, he says, what do you want now? He tries to make me eat. P3

This tension reflected the struggle between the patients not wanting to eat if they have no appetite and thus preserving their own sense of autonomy and the carers’ concern for their welfare which fuelled a desire to exert pressure on the patient to eat. The exacerbation of tension between the patients and their carers reflects the complex interplay between individual agency, resistance and relational dynamics within the carer-patient relationship.

My daughter keeps telling me you know ‘You're going to have to eat more.’ You know but I say why, I don't have an appetite so why would I force myself to eat it.” P2

Carers expressed their struggles with communication challenges, finding it difficult to broach the topic of their loved one's worsening condition, particularly regarding eating habits. The patient's resistance to eat which was also driven by a desire to preserve some autonomy, compounded these challenges and led to potential conflicts. This dynamic which was predominantly focused on eating highlighted the emotional strain experienced by both parties. In response, carers employed strategies to sidestep conflict, such as avoiding direct discussions about eating habits. These avoidance tactics reflected carers' acknowledgment of the emotional strain and their attempt to navigate the complexities of the carer-patient relationship with sensitivity and understanding.

“That's when I noticed he was really getting worse, but you, you can't discuss that with him, he loses the rag, you know you don't tell him to do anything, he has to want to do it himself otherwise there's no point.” C1

This tension underscores the existential struggle between individual agency and external influence within the carer-patient dynamic. Moreover, carers recognised the importance of autonomy in maintaining the dignity of their loved one, yet their concern was palpable.

### 3.5 Social withdrawal and vulnerability

Both carers and patients described a significant reduction in social activities. This withdrawal from social life was attributed to the patient's illness. Social interactions around food become charged with existential significance, as patients perceived themselves as being watched or judged by others when they struggle to eat. This experience exacerbated their sense of withdrawing from social activities which involved food which impacted their sense of belonging and connectedness in social gatherings.

"…sometimes you don't want to be out because I don't want to be spoiling anybody else's night, you know. Being picky, you know" P1

Patients’ reluctance to engage with social activities which involved food reflected more than just a physical limitation; it represented a disruption in their ability to connect with the world and participate in a key activity which culturally represents vitality and togetherness. Withdrawal from eating together reflected a social withdrawal from a fundamental aspect of human existence leading to a sense of disconnection from their surroundings and extended family and friends. Patients expressed a profound sense of futility and resignation in their engagement with food which impacted multiple domains of their lives and collectively pointed towards a deeper existential struggle with the meaning and purpose of their life.

"You know when I was eating properly, I loved going out, we'd have went out at the weekend or even during the week for tea, but now I wouldn't even dream of going out anywhere." P2

Carers’ discomfort during meals and social outings stemmed from a heightened sense of helplessness in the face of recognition of their loved one's struggle and their own inability to alleviate their suffering or provide help. From the carers’ perspectives there was a responsibility for their loved one's well-being and an obvious tension between their efforts to reconcile their desire to help with the recognition of their own limitations and the harsh realities of their loved one's illness.

"When we go out for dinner and meals and stuff, you can see in her face when she has ordered something to eat and she's hardly touching any of it. She is looking and you're feeling a bit awkward that she hasn't touched anything on her plate. I think she finds that a bit awkward in a way" C2

Carers expressed their efforts to encourage their loved one to participate in social activities, they recognised the importance of social connection for their loved one's quality of life and strived to support them in accessing these opportunities. Carers demonstrated efforts to understand their loved one's personal lived experience and a commitment to supporting them.

"Well, the restrictive side of it has been … well going out is a problem. I have to work on her to persuade her to get in the car and go out” C3

The carers desire to engage in social activities with their loved one reflects a recognition of the importance of maintaining and nurturing their relational bonds through shared experiences and activities, even in the face of frustration and challenges.

### 3.6 Psychological strain amidst lack of information about cachexia

All factors noted in the preceding group experiential themes contributed to the psychological impact of renal cachexia, however this section specifically illustrates how the psychological strain amidst lack of information of renal cachexia had a direct effect on patients and carers. As carers engaged in a shared journey with their loved one the interconnected nature of caregiving was apparent. Carers expressed a deep sense of empathy and compassion for their loved one and were innately attuned to their suffering, however in response they vicariously suffered and expressed feelings of helplessness, guilt, and existential despair.

“I went to bed many a night crying… watching him [decline], very hard” C1

The carers’ expression of strength and stoic determination to carry on despite emotional challenges reflects their commitment to fulfilling their role as a source of support for their loved one. This determination was rooted in a deep sense of responsibility and love for their loved one, which drove them to prioritise their well-being above their own emotional needs. Carers appeared to feel pressure to maintain a sense of composure and strength, believing that it is not permissible for them to openly express emotional anguish or vulnerability.

“I take the approach in my mind that I'm not going to let myself become an emotional wreck, because of this situation.”C3

Renal cachexia had a psychological impact on patients’ self-perception, sense of identity and purpose. Patients questioned the meaning and significance of their existence in the context of their limited mobility and functionality in their experience of living with the syndrome.

"You know, I just, see myself as a really old woman, I don't buy myself clothes or anything, you know, I find myself going through everything. Why should do this? Because I can't go anywhere. So, what's the point?" P2

Patients and carers expressed a mix of frustration and sadness, particularly in response to weight loss, lack of appetite and reduced mobility. Male interviewees were more inclined to express frustration and anger, female patients and carers' sadness and anxiety. Uncertainty was common for carers and patients which was underpinned by a sense of helplessness as to what to do in response to weight loss and/or reduced appetite.

“And it just always just seems to be going down and going down and going down. My weight… [I] don't know when it's going to stop.” P3

“There's not really much you can really do to help. If she doesn't feel like eating… You can't really force her to eat something that she doesn't want to do.” C2

None of the patients or carers interviewed had ever heard of renal cachexia and therefore had no awareness or understanding of the impact or how to manage this condition. Patients and carers attributed exhaustion, appetite changes and weight loss to the effects of dialysis and other co-morbidities.

“The fact that I am on dialysis, and I do have heart problems, I get tired easily. And I feel like I can't join in the way other people would.” P4

“Then she went back onto dialysis. Her appetite picked up again. But then, like I said, it diminished then, after a while being on dialysis.” C2

Overall, patients and carers struggled to make sense of the symptoms they or their loved one encountered which led to feelings of confusion, uncertainty, and a sense of unease as they navigated their lived experience with renal cachexia without a clear framework for understanding.

## 4 Discussion

This is the first study to explore the lived experience of renal cachexia for patients and carers through the lens of interpretive phenomenological analysis. While current research in renal cachexia is largely based on anatomical, physiological and pathological issues (Reid et al., 2013) this study uniquely underscores the complex interplay between physical decline, social and psychological impact for patients and carers.

Improving outcomes associated with cachexia such as loss of muscle mass and functionality through adequate nutritional intake are important but equally important is optimally managing the holistic impact of cachexia (McKeaveney et al., 2020, Arends et al., 2021, Reid et al., 2010, Blair et al., 2022, Blair et al., 2022, Hadzibegovic et al., 2020). This analysis reveals that weight loss in the context of renal cachexia is not only a physical manifestation but also an emotionally charged experience for both patients and carers. Similarities from our analysis can be found in cancer cachexia (Reid et al., 2009, Reid et al., 2010) and cardiac cachexia (Carson et al., 2022) which indicates the commonalities in lived experience for patients and carers across chronic illness. Cachexia evidently impacts mental health, emotions, and social interactions, therefore psychosocial support should be a prominent consideration in developing a holistic approach to multimodal interventions for cachexia management (McKeaveney et al., 2020, Amano et al., 2022, Reid et al., 2010, Blair et al., 2022, Blair et al., 2022, Hopkinson, 2021). Furthermore, the multifaceted nature of the challenges faced in eating for patients and 'assisting to eat' for carers, stresses the need for education in how to provide tailored dietary support and strategies to enhance outcomes for those experiencing renal cachexia.

Patients with renal cachexia and their carers navigate a challenging landscape marked by physical limitations, altered routines, and lack of information about renal cachexia. In the first mixed methods study eliciting the views of health care practitioners about renal cachexia, McKeaveney et al., (McKeaveney et al., 2020) reported that healthcare professionals recognised that a lack of standards of care or guidelines for the treatment of renal cachexia made current practice wide‐ranging. It was acknowledged by healthcare professionals that “information material [relating to renal cachexia] is scarce or absent” and this lack of information can mean that individuals with end-stage kidney disease are “very frightened” about changes in appetite that may be correlated with cachexia (McKeaveney et al., 2020). Healthcare professionals endeavour to deliver person-centred care, however, to do so in renal cachexia, patients and carers experiences and existential concerns must be understood (Reid et al., 2013, McKeaveney et al., 2020, Vélez and Ramasco, 2006).

In a related study, based on the lived experience of patients with cachexia and their carers, authors found that the lack of information resulted in family members feeling misinformed (Reid et al., 2010). It is evident through our study that the lack of evidence‐based guidelines contributes to the lack of awareness of the syndrome which negatively impacts on both those experiencing renal cachexia and their carers. Transparent information is necessary to strengthen the confidence of both patients experiencing cachexia and their significant others through understanding (Reid et al., 2010). Individuals with renal cachexia and their significant others require honest and problem-centred communication tailored to the disease stage and specificity. Research is needed in understanding patient and carer distress related to renal cachexia and ways to enhance communication which promotes and enables therapeutic effectiveness.

It is evident that food and eating are fundamental aspects of quality of life which are severely impacted by renal cachexia. Similar to cachexia in other chronic diseases, the emotional toll of eating challenges and social withdrawal which accompany early satiety and lack of appetite, lead to a loss of self and fractured personhood (Reid et al., 2009, Reid et al., 2010, Millar et al., 2013, Bland et al., 2021, Krysztofiak et al., 2020). However, this remains an under-diagnosed and undertreated condition which has a devastating impact on psycho-emotional and social domains (Reid et al., 2009, Reid et al., 2010, Millar et al., 2013, Bland et al., 2021, Krysztofiak et al., 2020). Findings in the study by McKeaveney et al. (McKeaveney et al., 2020) suggest that healthcare professionals deemed the most important factors when treating renal cachexia to be an improvement in quality of life (69 %) and relieving family distress (41 %). Considering how our study supports existing evidence, integrating a quality of life assessment into clinical practice is essential to measure and respond as to how cachexia impacts on various aspects of a person's life, including emotional well-being, social interactions, and daily functioning (McKeaveney et al., 2020, Arends et al., 2021, Reid et al., 2010, Blair et al., 2022, Blair et al., 2022, Hadzibegovic et al., 2020). Through gathering data on psychosocial outcomes including depression, anxiety, social isolation, and the ability to engage in daily activities, this could help to quantify the emotional and social burden of cachexia (McKeaveney et al., 2020, Amano et al., 2022, Reid et al., 2010, Blair et al., 2022, Blair et al., 2022, Hopkinson, 2021).

The evolving awareness of the significance of weight loss all contribute to feelings of fear, worry, helplessness and the inter-personal tensions in caring for a loved one with renal cachexia. Therefore, symptom identification and amelioration are understandably a high priority. Carers grapple with frustration, emotional strain, and the reciprocal impact on their physical well-being yet with a sense that they feel these emotions are not warranted and given due consideration. The witnessing of a loved one's suffering emerges as a profound challenge. Multi-perspective designs, such as this study involving carers, are necessary given that it is increasingly recognised that the experience of living with chronic disease “is not solely located within the accounts of those with the diagnosis” (Larkin et al., 2019), (p.182). This study supports existing research and policy documents on end-stage kidney disease which emphasise that patient ‘and’ carers input should be used to inform person-centred care delivery in kidney disease ((Vélez and Ramasco, 2006, Shi et al., 2022, European Kidney Health Alliance (EKHA) 2015, UK Kidney Association (UKKA) 2022, Kidney Care UK (KCUK) 2024, Department of Health 2005)). This study highlights the critical role carers play in the management of renal cachexia which underscores both the needs of the patients and the challenges faced by carers, there is a need for responsive interventions to consider the complexity of the carer dyad.

These findings emphasise the need for holistic support, including emotional and psychological aspects, in the care of individuals with renal cachexia. Although the refractory nature of their disease may make this difficult this does not mean that this syndrome should be addressed by silence. Rather there is a critical requirement for measures to identify and interventions to help patients and carers manage this better, by co-designing holistic support that both recognises and responds to the needs identified in our study. As such, there is an urgent need for further studies that could help to interpret these experiences, and thereby inform clinical practice guidelines.

## 5 Limitations

The findings presented within this paper must be interpreted taking into consideration the study's limitations. Most importantly, data from this study characterise patients’ and their carers’ perceptions of the experience of renal cachexia, no healthcare professional sample was recruited within this study, thus data from this perspective was not obtained. The interpretive phenomenological analysis method, although rigorous, involves a small sample size and degree of subjectivity that could influence the interpretation of the data and limit the generalisability of the results. The research was conducted within two healthcare trusts in the United Kingdom, which may affect the transferability of the findings to other regions or healthcare settings with different practices and support systems.

## 6 Conclusions

This study has formed an idiographic contribution to understanding the lived experience of patients with renal cachexia and their carers. The lived experience of cachexia encompasses appetite loss, functional decline, weight loss as a visual metaphor of concern, social withdrawal, and vulnerability. Additionally, patients and carers face the emotional toll of eating challenges and psychological strain amidst a lack of information. In response, care delivery should recognise and respond to the distinctive needs of this population. Future research is urgently required to develop models of holistic support which could help patients and carers cope with the impact of renal cachexia and optimally manage this syndrome within the family unit. In addition, further research is required to work towards uniform criterion for screening, diagnosis and optimum therapy to update and create relevant clinical guidelines and thereby provide much needed information to patients and carers (Reid et al., 2018, McKeaveney et al., 2021, McKeaveney et al., 2021, McKeaveney et al., 2020, Reid, 2014, Kokura et al., 2017). Finally, when triangulated with existing studies relating to renal cachexia (Reid et al., 2018, Reid et al., 2015, McKeaveney et al., 2021, McKeaveney et al., 2021, McKeaveney et al., 2020), this research has international applicability to increase specific understanding of the lived experience of renal cachexia in comparison to cachexia in other chronic diseases.

## Data sharing statement

This is a great paper. Great work, authors. You've shared a powerful story on an important topic.

## Funding sources

No external funding.

## CRediT authorship contribution statement

**Carolyn Blair:** Writing – review & editing, Writing – original draft, Visualization, Validation, Software, Resources, Project administration, Methodology, Investigation, Formal analysis, Data curation, Conceptualization. **Joanne Shields:** Writing – review & editing, Validation, Methodology, Conceptualization. **Robert Mullan:** Writing – review & editing, Validation, Methodology, Conceptualization. **William Johnston:** Writing – review & editing, Validation, Methodology, Conceptualization. **Andrew Davenport:** Writing – review & editing, Validation, Methodology, Conceptualization. **Denis Fouque:** Writing – review & editing, Validation, Methodology, Conceptualization. **Kamyar Kalantar-Zadeh:** Writing – review & editing, Validation, Methodology, Conceptualization. **Peter Maxwell:** Writing – review & editing, Validation, Methodology, Conceptualization. **Clare McKeaveney:** Writing – review & editing, Validation, Methodology, Conceptualization. **Helen Noble:** Writing – review & editing, Validation, Methodology, Conceptualization. **Sam Porter:** Writing – review & editing, Validation, Methodology, Conceptualization. **David Seres:** Writing – review & editing, Validation, Methodology, Conceptualization. **Adrian Slee:** Writing – review & editing, Validation, Methodology, Conceptualization. **Ian Swaine:** Writing – review & editing, Validation, Methodology, Conceptualization. **Miles Witham:** Writing – review & editing, Validation, Methodology, Conceptualization. **Joanne Reid:** Writing – review & editing, Visualization, Validation, Supervision, Software, Resources, Project administration, Methodology, Investigation, Formal analysis, Data curation, Conceptualization.

## Declaration of competing interest

The authors declare that they have no known competing financial interests or personal relationships that could have appeared to influence the work reported in this paper.

## Appendix Supplementary materials

Image, application 1

Image, application 2
