# Supplementary material for: The lived experience of renal cachexia: An interpretive phenomenological analysis
Source: Int J Nurs Stud Adv. 2024 Aug 22;7:100235. doi: 10.1016/j.ijnsa.2024.100235 (PMC11426076; doi:10.1016/j.ijnsa.2024.100235)
Supplement: Supplementary file 1 [file mmc1.docx]

Supplementary File 2

Table 1: GRIPP2 short form

| Section and topic | Item |
| --- | --- |
| 1: Aim | This study was developed with advice and contribution from patient and carer representatives from Northern Ireland Kidney Patients Association and Northern Ireland Kidney Research Fund. Additionally, one individual experiencing chronic kidney disease in receipt of haemodialysis has been involved from inception and is a co-author which reflects his ongoing involvement at all stages of the research process. The aim of involvement was to ensure this research addressed real-world concerns and priorities of patients and the public. |
| 2: Methods | By participating in the study from its inception, our patient and public involvement partner helped to shape the aim and the topic areas in the interview guide, reviewed all participant facing materials and was involved in edits to the final paper and is a co-author. Contact with this individual was conducted regularly throughout the duration of the project via email and scheduled MS Teams meetings. |
| 3: Study results | Primary results and dissemination plan were discussed at a workshop in November 2023 with patient and carer representatives from Northern Ireland Kidney Patients Association and Northern Ireland Kidney Research Fund. Our patient co-author assessed the relevance of the findings and helped the team to develop the themes reported on the first draft of the findings and subsequent versions. |
| 4: Discussion and conclusions | The input of our patient and carer representatives collectively helped to shape the discussion and conclusion to be more relevant and practical, making the study more applicable to those it aimed to help. |
| 5: Reflections/critical perspective | Our patient and public involvement partners from Northern Ireland Kidney Patients Association and Northern Ireland Kidney Research Fund have worked with our research team over a significant period of years. The team's relationship with our partners have strengthened over the years, highlighting the importance of sustaining long-term collaborations with patient and public involvement partners.  There were limitations to our patient co-author’s participation. Due to research ethics requirements, this individual did not have access to the transcripts and could only comment on the deidentified data presented after analyses and was not trained in qualitative research, so could not comment on the methodological aspects of the study. |
